# Sleep and energy intake in early childhood

**DOI:** 10.1038/ijo.2014.50

**Published:** 2014-04-29

**Authors:** A Fisher, L McDonald, C H M van Jaarsveld, C Llewellyn, A Fildes, S Schrempft, J Wardle

**Affiliations:** 1 Health Behaviour Research Centre, Department of Epidemiology and Public Health, University College London, London, UK; 2 Department of Primary Care and Public Health Sciences, King’s College London, London, UK

**Keywords:** sleep, diet, child

## Abstract

**Background And Objectives:**

Shorter sleep is associated with higher weight in children, but little is known about the mechanisms. The aim of this study was to test the hypothesis that shorter sleep was associated with higher energy intake in early childhood.

**Methods:**

Participants were 1303 families from the Gemini twin birth cohort. Sleep duration was measured using the Brief Infant Sleep Questionnaire when the children were 16 months old. Total energy intake (kcal per day) and grams per day of fat, carbohydrate and protein were derived from 3-day diet diaries completed by parents when children were 21 months old.

**Results:**

Shorter nighttime sleep was associated with higher total energy intake (*P* for linear trend=0.005). Children sleeping <10 h consumed around 50 kcal per day more than those sleeping 11–<12 h a night (the optimal sleep duration for children of this age). Differences in energy intake were maintained after adjustment for confounders. As a percentage of total energy intake, there were no significant differences in macronutrient intake by sleep duration. The association between sleep and weight was not significant at this age (*P*=0.13).

**Conclusions:**

This study provides the first evidence that shorter nighttime sleep duration has a linear association with higher energy intake early in life. That the effect is observed before emergence of associations between sleep and weight indicates that differences in energy intake may be a mechanism through which sleep influences weight gain.

## Introduction

Obesity affects one in five children in the United Kingdom, programming a lifetime increase in risk of health problems.^1^ The direct and indirect economic costs of obesity are enormous and expected to increase exponentially over the next decade.^2^ Once gained, weight is extremely difficult to lose; early life prevention is therefore vital.

Sleep length has attracted attention as a novel risk factor for overweight and obesity in children,^3, 4, 5^ although effects are more mixed in adults.^3^ A meta-analysis of 11 studies (29 502 children aged 2–20 years) found a pooled odds ratio for obesity in those sleeping ⩽10  vs >10 h of 1.89 (95% confidence interval (CI)=1.46, 2.43).^6^ In the largest single pediatric study, involving 8274 Japanese 6 to 7 year olds, those who slept <8 h were almost three times more likely to be overweight than those who slept for >10 h.^7^ In the same cohort, sleep duration significantly influenced weight gain from 3 to 6 years.^8^ The studies in the review all used parent-reported measures of sleep, but one of the few pediatric studies to have used objective measures found that each additional hour of sleep at age 3–5 years was associated with a reduction in body mass index of −0.49 (95% CI=−0.01 to −0.96) at 7 years.^9^

There is increasing evidence that changes in eating behavior underlie weight differences between shorter and longer sleepers.^10^ In adults, acute sleep restriction has been shown to increase ghrelin and decrease leptin (hormonal regulators of energy intake), as well as alter glucose homeostasis and insulin sensitivity.^11, 12, 13^ Functional magnetic resonance imaging studies have also found increased brain activation in areas associated with food stimuli and reward following extreme sleep restriction (4 vs 8–9 h).^14,15^ During *ad libitum* access to food, controlled sleep restriction (<5 h) has been shown to increase energy intake and induce weight gain in healthy adults (0.82 kg over 5 days of sleep restriction).^16^ Importantly, controlled changes in sleep duration have also been found to alter energy intake and weight in 8–11-year-old children.^17^ However, sleep deprivation also causes physiological stress, which may itself alter energy-balance regulators. Furthermore, young children have less autonomy over their eating behavior and food environment,^18^ so findings from adults and older children may not generalize to very young populations. Studies that reflect habitual levels of sleep and eating behavior in young children are therefore important additional sources of information.^19^

There are some data from adults and adolescents suggesting differences in dietary composition, although results have been mixed. In two studies, very short sleepers (<6 h) had more erratic mealtimes and higher levels of snacking.^20,21^ In the Women’s Health Initiative, objectively measured nocturnal sleep was negatively associated with fat intake after adjustment for body mass index,^19^ and in the National Health and Nutrition Examination Survey, adults reporting 7 to 8 h of sleep also reported greater food variety and lower energy intake compared with adults reporting habitual short (5–6 h) or long (>9 h) sleep.^22^ Three adolescent studies found that shorter sleepers had higher carbohydrate intake,^23^ higher fat intake,^24^ higher consumption of energy-rich foods and lower fruit and vegetable intake,^25^ and in a study of Portuguese 5 to10 year olds, shorter sleepers had lower fruit and vegetable intake.^26^

We are not aware of any investigations of associations between habitual sleep and energy intake in early life, before associations with weight emerge. The aim of the present study was therefore to test the hypothesis that shorter sleep would be related to higher energy intake in early childhood.

## Participants and methods

The Gemini study has been described in detail previously.^27^ Briefly, it is a population-based cohort of young twins in the United Kingdom focusing on the determinants of early childhood weight trajectories. The total sample comprises 2402 families with twins born in England and Wales between March and December 2007 (36% of all live twin births). The present study analyzed data from 1303 families with complete sleep and dietary data (using data on 1 child from each family). Sleep data were collected when the twins were around 16 months old (mean 15.7, s.d. 1.1). Dietary data were collected at around 21 months (mean 20.7, s.d. 1.6). Weight data were available for a mean age of 17. 8 months (s.d. 3.7).

Gemini families are comparable to UK national twin statistics on sex, zygosity, gestational age and birth weight,^27^ although in common with other population-based samples, ethnic minorities and lower socioeconomic groups are under-represented, and participants are slightly healthier than average with respect to parental diet, smoking rates and body mass index.^27^ The majority (95%) were from a ‘white’ ethnic group. Parents who provided complete diet and sleep data were slightly older (34.6 vs 32.5 years), had slightly higher education levels and were more likely to be from a ‘white’ ethnic group (*P*s all <0.001). Informed written consent was provided by all parents. Ethical approval was granted by the University College London Committee of non-National Health Service Human Research.

Sleep was assessed using modified items from the Brief Infant Sleep Questionnaire for children, which has been shown to be reliable and is validated against actigraphy in children aged 5–29 months.^28^ Parents reported a number of aspects of their children’s sleep, including usual bed time and wake time, from which nighttime sleep duration was calculated. Daytime nap duration was also reported. In a subsample of 40 Gemini parents, 1-week test–retest reliability of the sleep questionnaire was excellent (intraclass correlation 0.89; 95% confidence interval (CI): 0.76–0.95 for sleep duration). Given the lack of consensus on the definition of ‘short’ vs ‘adequate’ sleep in the literature, we identified five groups: <10 h a night, 10–<11 h a night, 11–<12 h a night, 12–<13 h a night and ⩾13 h a night, which would also allow a test of a linear association.

Dietary intake was estimated from diet diaries. Parents were asked to record everything their children ate and drank for 3 days (including one weekend day) in simple diaries provided to them. Detailed instructions were provided with the diary, along with pictorial food and portion guides. The diaries were coded by researchers at Experts in Nutrition at the Medical Research Council Human Nutrition Research. The coding process involved each food in the diary being entered into the nutrient database and matched, and then a portion size estimate added. Dietary variables examined were: total energy intake (mean daily kcal over 3 days), mean daily grams of fat and carbohydrate and protein. The proportion of total energy from fat, carbohydrate and protein was calculated using the Atwater conversion factors (protein: 4 kcal g^−1^ fat: 9 kcal g^−1^ and carbohydrate: 3.75 kcal g^−1^).^29^

Potential confounders included child age, sex, weight and maternal education. Because daytime naps were common in our child sample, nap duration was also considered in case shorter nighttime sleepers were compensating during the day. Parents reported their highest education level that was categorized as: lower (compulsory schooling only; 29% of the sample), middle (some additional school or vocational examinations; 22%) and higher (university education; 49%). Children’s age (in months) at sleep questionnaire completion and at diet diary completion were recorded. The child's weight in kilograms (kg) were converted to age- and sex-adjusted standard deviation scores relative to the UK 1990 reference data.^30^ Gemini parents provide ongoing 3-monthly weight measurements, so weight data close to sleep data were selected, and were available for 1094 of the sample (84%). The exact age of the weight measurement was included as a potential confounder in analyses using weight data.

### Analyses

Dietary and sleep data were normally distributed. Univariate comparisons were made using *χ*^2^ or analysis of variance where appropriate. There were no significant interactions between sleep or energy intake and gender, so data from boys and girls were combined. Comparison of dietary variables between sleep groups adjusting for confounders were carried out using analysis of covariance using tests for linear associations (polynomial contrasts). Contrast estimates for difference from analysis of covariance models are presented. Models were run with and without weight as a confounder and results were the same, so to maximize sample size data from analysis of covariance, models without adjustment for weight are presented. Analyses were repeated using 24 h sleep and results did not change; therefore, associations with nighttime sleep (adjusting for daytime nap) are reported throughout. Data were analyzed using SPSS Version 18 (IBM, Hampshire, UK) and *α* set at *P* <0.05.

## Results

Participant characteristics by nighttime sleep duration group are shown in Table 1. There were no significant differences in daytime nap duration by nighttime sleep duration. Weight (kg) and weight standard deviation scores did not differ significantly by nighttime sleep group (*P*s all >0.05). There were slightly more boys in the shorter-sleeping groups and slightly more girls in the longer sleeping groups (*P*<0.001), and maternal education levels varied across sleep groups (*P*<0.001). There were very slight but significant differences in the age of diet diary completion across sleep groups, with both the shortest and longest sleeping group being slightly younger than the other groups (*P*<0.001).

Unadjusted associations between sleep and energy intake are shown in Table 2. Total energy intake was significantly higher in shorter-sleeping groups, with a linear association across sleep durations (*P*=0.005), with children who slept <10 h a night consuming an average of 105 kcal per day (95% CI: 17–193 kcal per day) more than those sleeping for ⩾13 h a night. Protein intake (g per day) was not significantly associated with sleep duration (*P*=0.20). Although differences were small, intake of fat (g per day) and carbohydrate (g per day) were associated with sleep, with shorter sleepers consuming more (*P*’s for linear trends *P*=0.02 and 0.008, respectively). Expressed as a percentage of total daily calories, however, there were no significant differences in macronutrient composition by sleep duration (*P*s all >0.05).

After adjusting for confounders, energy intake remained significantly higher in shorter-sleeping groups and the association was linear (contrast estimate for difference 71; 95% CI: 19–123 kcal per day; difference between longest and shortest sleepers 102 kcal per day, *P*=0.008).

Associations between total grams of fat and carbohydrate also remained significant after adjustment. For fat, the linear contrast estimate for difference was 3 g per day (95% CI: 0.5–6; *P*=0.02), and for carbohydrate, the estimate for difference was 10 g per day (95% CI: 2–17; *P*=0.01).

## Discussion

This study provides the first evidence that shorter sleep is associated with higher energy intake in early childhood. The associations were observed in a population sample reporting habitual diet and sleep, and before any association between sleep and weight. These results address a significant gap in the literature. The need to examine sleep and diet in a naturalistic setting has been highlighted as a research priority.^5^ This is particularly pertinent in the light of concern that the sleep-weight ‘bandwagon’ is progressing in the absence of evidence for mechanisms.^4,31^

This is the first study to test for a linear relationship between sleep duration and energy intake in early childhood. The few existing pediatric or adolescent studies have generally categorized sleep into ‘short’ vs ‘long’ sleep duration using a variety of cut-points.^23, 24, 25, 26^ Our results add to the findings from these studies by demonstrating that associations exist across the spectrum of sleep duration. Given that we did not observe a sleep and weight association at this young age, this reduces the possibility that already bigger children are simply eating more.

It is not possible to tell whether the increased energy intake was a physiological effect of shorter sleep or the result of shorter-sleeping children being awake longer and having more time to eat. A number of experimental studies in adults show that acute sleep restriction can influence appetite hormones, with increases in ghrelin and decreases in leptin after short sleep, as well as increases in hunger.^11,13^ The hypothesis that more time awake simply results in more time to eat is not fully supported in adult studies, where both ‘too short’ and ‘too long’ sleepers often have higher body mass index, although some confounding by psychological problems like depression and anxiety is likely.^31^ However, relationships between sleep and eating patterns in young children should be explored.

There have been few comparable studies in children, and none in children under 5 years of age. One study in adolescents found that those sleeping <8 h a night consumed approximately 10% more calories than the longer sleeping group, and had higher fat intake.^24^ An experimental study of school aged children found that when children decreased their sleep duration to 7 h per night, their daily energy intake increased by 8% (134 kcal per day), and they weighted 0.24 kg more after 1 week.^17^ In the present study, shorter sleepers (<10h) consumed 50 kcal more each day than children obtaining an optimal amount of nighttime sleep (11–<12h). Although this difference is relatively small, it equates to around 5% of the mean daily energy intake in the sample, and is proportionately comparable to that observed in studies of older children and adolescents.^17,24^

In our study, intake of fats and carbohydrates were significantly higher in shorter sleepers as would be expected from the higher energy intake, but dietary composition did not differ across the spectrum of sleep duration; rather, children who slept for shorter periods at night tended to eat more overall. Although we found no evidence for differences in macronutrients when considered as a percentage of total energy intake, results from studies across various age groups have found that shorter sleep is associated with higher consumption of (or preference for) fat,^19,24,32,33^ higher carbohydrate intake and lower intake of fruit and vegetables.^25,26,34^ Shorter sleep has also been linked with increased snacking and eating at more unconventional hours.^20,21,24,35^ In early childhood, the context of feeding is largely determined by parents, so the impact of sleep on the amount, composition and distribution of energy intake may change in childhood and adolescence when the individual has more dietary autonomy.

One emerging perspective is that a longer duration of wakefulness carries a metabolic cost that initiates a series of biobehavioral changes designed to encourage energy intake and conservation.^36^ Our results are consistent with the idea that there is a behavioral adaptation to short sleep in early life. It is potentially an adaptive response to the metabolic cost of being awake for longer, but in a modern environment that encourages excess consumption and sedentary behavior, these changes may become maladaptive. While we observed relatively small differences in energy intake across the five sleep groups, small sustained changes over time have the potential to shift the population distribution of overweight and obesity.

### Strengths and limitations

A strength of this study is the use of detailed dietary data in a population-based cohort. Examining associations with sleep before relationships between sleep and weight emerge strengthens the argument for a causal role, although cannot confirm causality. Reliance on parental report is a common limitation of larger-scale population studies. Future work could assess sleep objectively using accelerometery, but ‘measuring’ diet objectively in a large sample is challenging. However, better measures would most likely increase the strength of the sleep and energy intake association. While we observed differences across a wide range of sleep durations, it is important to note that sleeping <10 or >13h a night is not typical at this age, and as a result, the number of participants in these groups were small. However, the linear trend was maintained when sleep duration was categorized into three larger groups (<11, 11–<12 and ⩾12h). There are other potential explanatory factors that were not measured in this study, such as parental eating patterns and activity levels. Our sample was drawn from a twin cohort, albeit we only used data from one child per family. There is no reason to expect that sleep and energy intake associations would be different to singletons, particularly as aspects of sleep architecture such as duration and disturbance are very similar,^37^ but this possibility cannot be ruled out. We observed socioeconomic and ethnic differences between families who provided full data and those who did not, but the magnitude of the differences was small, and again, there is no reason to suspect that associations between sleep and energy intake would differ by socioeconomic group. However, these associations should be examined in larger samples of ethnically diverse populations.

## Conclusion

Shorter nighttime sleep duration is associated with higher energy intake in early childhood before differences in weight have emerged. A higher energy intake is a plausible mechanism through which shorter sleep contributes to adiposity in early life, and parents should be aware that their shorter-sleeping children may be prone to consume more.

## Figures and Tables

**Table 1:** Participant characteristics by nighttime sleep duration (mean (s.d.), unless stated otherwise)

*Nighttime sleep duration*	*<10 h*	*10*–*<11* *h*	*11*–*<12 h*	*12*–*<13 h*	*⩾13 h*	P*-value*
Number (total *n*=1303)	29	136	602	458	78	
Age (months) at the time of sleep record	15.5 (1.1)	15.8 (0.9)	15.7 (1.1)	15.8 (1.2)	15.7 (1.1)	0.58
Age (months) at the time of diet diary completion	20.4 (0.7)	20.8 (1.0)	20.7 (1.2)	20.7 (1.4)	20.1 (1.3)	<0.001
Sex (%) boy/girl	52/48	58/42	54/46	42/58	43/57	<0.001
Maternal education (%) lower/mid/higher)	48/21/31	38/17/45	26/23/51	29/20/51	35/33/32	<0.001
Daytime nap duration (h)	2.0 (0.9)	1.8 (0.7)	1.8 (0.6)	1.9 (0.6)	1.8 (0.8)	0.80
Weight (kg)^a^	10.6 (1.4)	11.0 (1.8)	10.9 (1.5)	11.0 (1.6)	10.6 (1.4)	0.13
Weight SDS^a^	−0.1 (1.0)	0.1 (1.2)	−0.1 (1.1)	0.1 (1.1)	−0.2 (1.1)	0.13

Abbreviation: SDS, standard deviation scores.

a Weight comparisons are adjusted for exact age when weight data were provided; weight data were available for 84% of the sample.

**Table 2:** Dietary data by nighttime sleep duration

*Nighttime sleep duration*	*<10 h*	*10–<11 h*	*11–<12 h*	*12–<13 h*	*>13h*	P*-value (linear trend)*
Number (total *n*=1303)	29	136	602	458	78	
Total energy intake (kcal per day)	1086.8 (208)	1053.4 (43)	1037.4 (182)	1029.1 (187)	981.5 (203)	0.005
Fat (g per day)	43.7 (11.2)	43.6 (10.3)	42.2 (10.1)	42.0 (10.2)	39.2 (10.1)	0.02
Carbohydrate (g per day)	141.5 (31.3)	133.3 (27.2)	132.8 (26.7)	130.8 (25.9)	126.9 (29.5)	0.008
Protein (g per day)	40.8 (10.2)	40.2 (9.2)	39.9 (8.4)	40.3 (8.8)	38.3 (8.2)	0.20
Fat (%)	36.0 (5.3)	37.2 (5.0)	36.5 (5.0)	36.5 (4.7)	35.9 (4.8)	0.66
Carbohydrate (%)	49.0 (6.6)	47.6 (5.8)	48.1 (5.6)	47.8 (5.4)	48.5 (5.7)	0.79
Protein (%)	15.0 (2.6)	15.3 (2.3)	15.4 (2.2)	15.7 (2.2)	15.7 (2.3)	0.06
